# Identifying the Relationships between Water Quality and Land Cover Changes in the Tseng-Wen Reservoir Watershed of Taiwan

**DOI:** 10.3390/ijerph10020478

**Published:** 2013-01-28

**Authors:** Hone-Jay Chu, Chun-Yu Liu, Chi-Kuei Wang

**Affiliations:** Department of Geomatics, National Cheng Kung University, No. 1, University Road, Tainan City 701, Taiwan; E-Mails: cute_chunyu_0527@hotmail.com (C.-Y.L.); chikuei@mail.ncku.edu.tw (C.-K.W.)

**Keywords:** remote sensing, NDVI, water quality, land cover change, regression

## Abstract

The effects on water quality of land use and land cover changes, which are associated with human activities and natural factors, are poorly identified. Fine resolution satellite imagery provides opportunities for land cover monitoring and assessment. The multiple satellite images after typhoon events collected from 2001 to 2010 covering land areas and land cover conditions are evaluated by the Normalized Difference Vegetation Index (NDVI). The relationship between land cover and observed water quality, such as suspended solids (SS) and nitrate-nitrogens (NO_3_-N), are explored in the study area. Results show that the long-term variations in water quality are explained by NDVI data in the reservoir buffer zones. Suspended solid and nitrate concentrations are related to average NDVI values on multiple spatial scales. Annual NO_3_-N concentrations are positively correlated with an average NDVI with a 1 km reservoir buffer area, and the SS after typhoon events associated with landslides are negatively correlated with the average NDVI in the entire watershed. This study provides an approach for assessing the influences of land cover on variations in water quality.

## 1. Introduction

Land use and land cover changes, associated with human activities and natural factors, compromise many ecosystem services in a watershed [1,2]. For example, forestland converted to agricultural or urban land may have increased erosion, runoff, and flooding [3]. Changes in land use and land cover interact with anthropogenic and natural drivers to affect the water quality of watersheds. Studies have used environmental and landscape data to examine the relationships between land use and land cover changes and suspended sediments [4,5,6,7] and nutrients [1,7,8,9,10]. Ahearn *et al*. showed that land use and land cover exert the greatest control over water quality in the Cosumnes Watershed, California [7]. The percentage of agricultural coverage had a significant influence on nutrient loading. Sliva and Williams used multivariate analysis to determine whether there was a correlation between water quality and landscape characteristics within the local Southern Ontario watersheds in Canada. They compared the influences of buffer zones and whole catchment landscape characteristics on water quality [9]. Li *et al*. showed the impact of land use and land cover on the water quality in the Upper Han River basin, China [10]. The correlation and regression analysis indicated that water quality was significantly related to vegetated coverage. 

Water quality is controlled by numerous anthropogenic and natural factors [7]. The quality of receiving waters is affected by human activities in a watershed by point sources, such as wastewater treatment facilities, and non-point sources, such as runoff from urban areas and farmland [11]. Understanding non-point source pollution requires an understanding of how particular land covers influence water quality within a watershed. The extent that land covers hierarchically affect water quality at space-time scales is a key question. The most widely used land cover index in this context is the normalized difference vegetation index (NDVI), which is a function of red and near-infrared spectral bands [12]. On a regional scale, multi-temporal NDVI images are practical for monitoring vegetation dynamics. The multi-temporal NDVI is useful for classifying land cover and detecting the dynamics of vegetation [13,14]. However, major changes in the NDVI are noted near landslides that were induced by disturbances in Taiwan [15,16]. For example, a typhoon is one of major natural disturbances to land cover. Sequent typhoons and rainstorms cause abnormal destruction to the vegetation; this destruction is influenced by rainfall distributions and typhoon paths [15]. 

The NDVI data were derived from SPOT satellite images in the Tseng-Wen Reservoir Watershed, Taiwan, before and after Typhoon Morakot and several other large typhoons (e.g., Typhoon Mindulle in 2004, Haitang in 2005, Sepat in 2007, Kalmaegi in 2008, and Fanapi in 2010) [17] to identify the changes to land cover. To represent land use and land cover change, an evaluation of multiple NDVI spatial scales was conducted. The study identified and delineated the relationships between temporal variations of the NDVI and water quality in the study area.

## 2. Materials and Methods

### 2.1. Study Area

**Figure 1:**
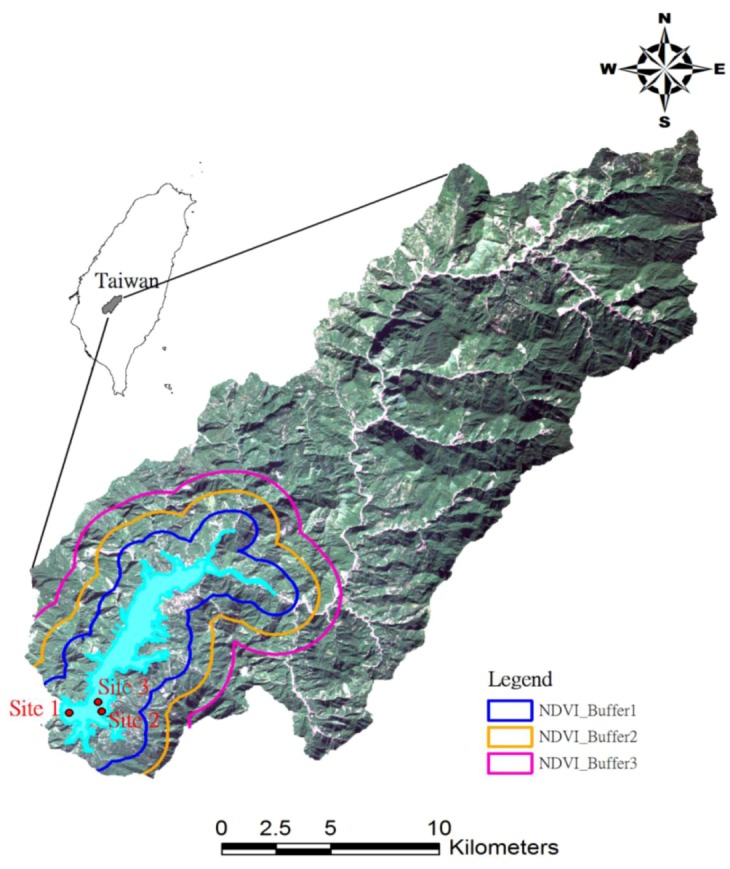
Location of Tseng-Wen Reservoir watershed and water quality sampling stations.

The Tseng-Wen Reservoir is a multipurpose reservoir designed for flood control, hydroelectric power generation, irrigation, water supply, recreation, and flow augmentation. The storage capacity of the Tseng-Wen reservoir is 608 × 10^6^ m^3^, but its effective water storage is 490 × 10^6^ m^3^, and the hydroelectric plant capacity is 50 MW. The Tseng-Wen Reservoir Basin is located in the upstream area of the Tseng-Wen River system in Chiayi County (Figure 1). The entire watershed area of this river basin is 1,176 km^2^, in which the Tseng-Wen Reservoir watershed covers 481 km^2^. The average slope of this river basin is approximately 1/57. Average rainfall in this watershed area is approximately 3,000 mm per year and annual average temperature is about 23.4° Celsius. Rich soil in the watershed is suitable for fruit and tea farms. Agriculture has restricted near the reservoir but tourism has developed in recent years. 

Numerous major typhoons have struck Taiwan, such as Typhoon Mindulle in 2004, Haitang in 2005, Sepat in 2007, Kalmaegi in 2008, Morakot in 2009, and Fanapi in 2010 [17]. Especially, Typhoon Morakot struck Taiwan from 7 to 9 August, 2009, and produced record-breaking rainfall and catastrophic damage in Southern Taiwan. The typhoon produced copious amounts of rainfall peaking at 2,777 mm*.*

### 2.2. Satellite Images

Multi-temporal Système Pour l'Observation de la Terre (SPOT) satellite images obtained after typhoons in 2001, 2003–2005, and 2007–2010 were used to quantify land cover changes in our study. Details for the dates are listed in Table 1. For atmospheric correction, Fast Line of Sight Atmospheric Analysis of Spectral Hypercube (FLAASH) is applied to correct the visible and near-infrared wavelengths in the satellite images [18]. Then, the NDVI maps were derived from the SPOT images taken in 2001 with a 20 m resolution, and with a 10 m resolution in 2003–2010. Moreover, the SPOT images were classified using supervised classification by the software package ERDAS IMAGINE. Land-use types were classified into the following six categories: forested land, built-up land, landslide, grassland, water, and bare land [19]. The reference maps were the aerial photographs by the Aerial Survey Office, Forestry Bureau, Taiwan.

**Table 1:** Mean andstandard deviation of NDVI maps during 2001–2010.

	Date	Mean	SD
2001	2001/10/22	0.585	0.205
2003	2003/12/30	0.704	0.163
2004	2004/12/29	0.600	0.171
2005	2005/11/05	0.663	0.191
2007	2008/01/05	0.563	0.209
2008	2008/11/12	0.686	0.171
2009	2009/11/01	0.437	0.218
2010	2010/12/27	0.494	0.221

SD: standard deviation.

### 2.3. Water Quality Data

Seasonal time series of water-quality data monitored in the reservoir were obtained from Taiwanese EPA Web sites [20]. The water quality data observed at three stations were obtained from 2001 to 2010 and data sampling frequency was three months. The sampling sites are shown in Figure 1. The water-quality variables, such as nitrate-nitrogen (NO_3_-N), suspended sediments (SS), chemical oxygen demand (COD), dissolved oxygen (DO), total phosphorus (TP), and turbidity, were derived for analysis. The variables are used as general indicators of water quality*.* For example, the COD is commonly used to measure the amount of organic compounds in water. As the DO in water drops below a threshold, aquatic life is under stress. The presence of high nitrates and TP concentrations in water indicates possible pollution of the water. Turbidity is the haziness of a fluid caused by the SS, which are solid particles usually transported by flowing water.

### 2.4. Regression Model

Reservoir water chemistry was sampled at the outlets and downstream of the reservoir every three months. We refer to the water samples after typhoons in the fourth season and acquire annual values each year. We ran a series of models to examine the correlations between land-cover and the water quality variables.

Our basic statistical tool was stepwise multiple linear regression, with backward selection of variables and *p =* 0.1 to enter or remove variables. Cases with missing data were excluded. Statistical analyses were done using SPSS 10.0. The dependent (response) variables are NO_3_-N and SS concentrations that are selected from the high-NDVI correlated water quality factors. The variable details are shown in Section 3.2. In addition, the independent variables are average NDVI at various spatial scales such as average NDVI in whole watershed, average NDVI in reservoir 1 km, 2 km and 3 km buffer zones, can be represented as:

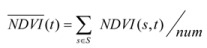
(1)
Where *NDVI*(*s,t*) represent NDVI value varies with space *s* and time *t*; 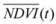 is average NDVI in whole watershed or reservoir buffer zone during time *t*; *S* is the domain set that defined as whole watershed or reservoir buffer zone; *num* is the number of the pixels in the set *S*.

## 3. Results and Discussion

### 3.1. Temporal Land Use and NDVI Changes

**Figure 2:**
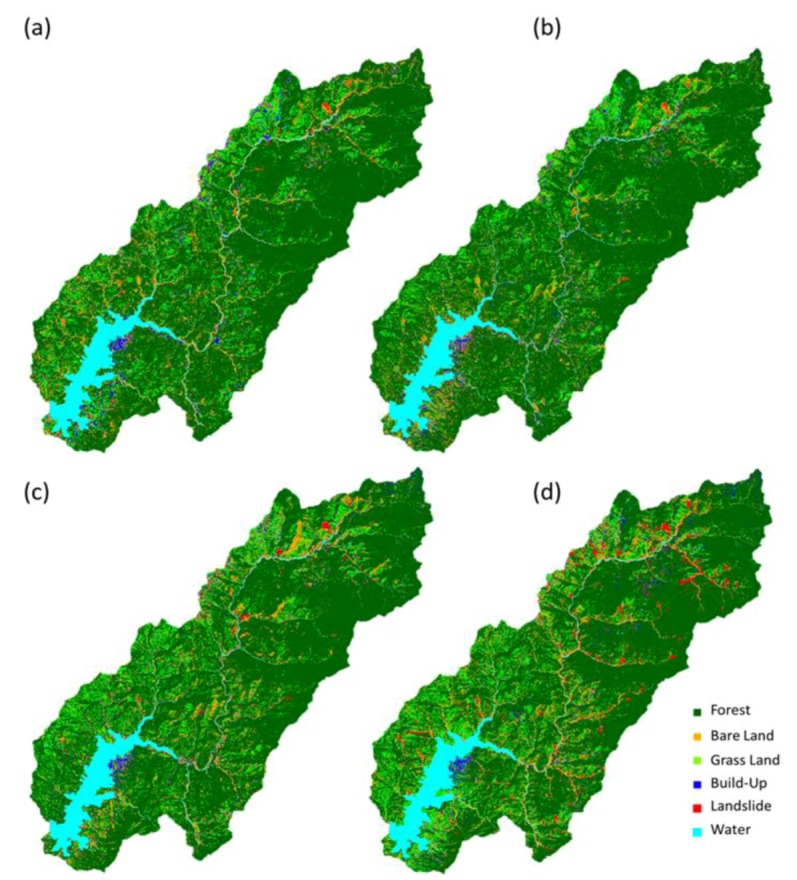
Land use classification in (**a**) 2001, (**b**) 2004, (**c**) 2007 and (**d**) 2010.

**Figure 3:**
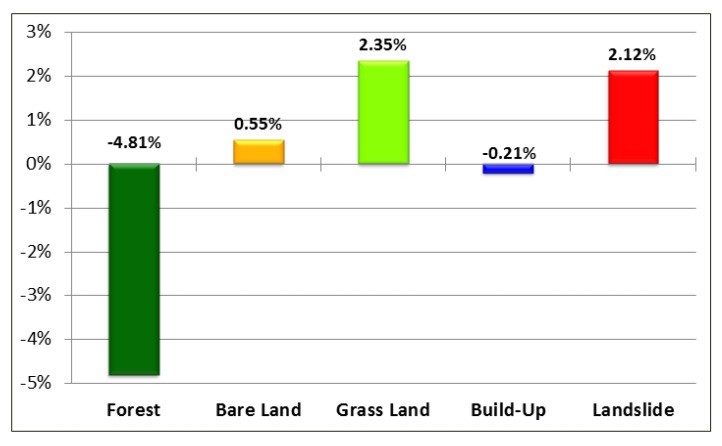
Land use change percentage from 2001 to 2010.

**Figure 4:**
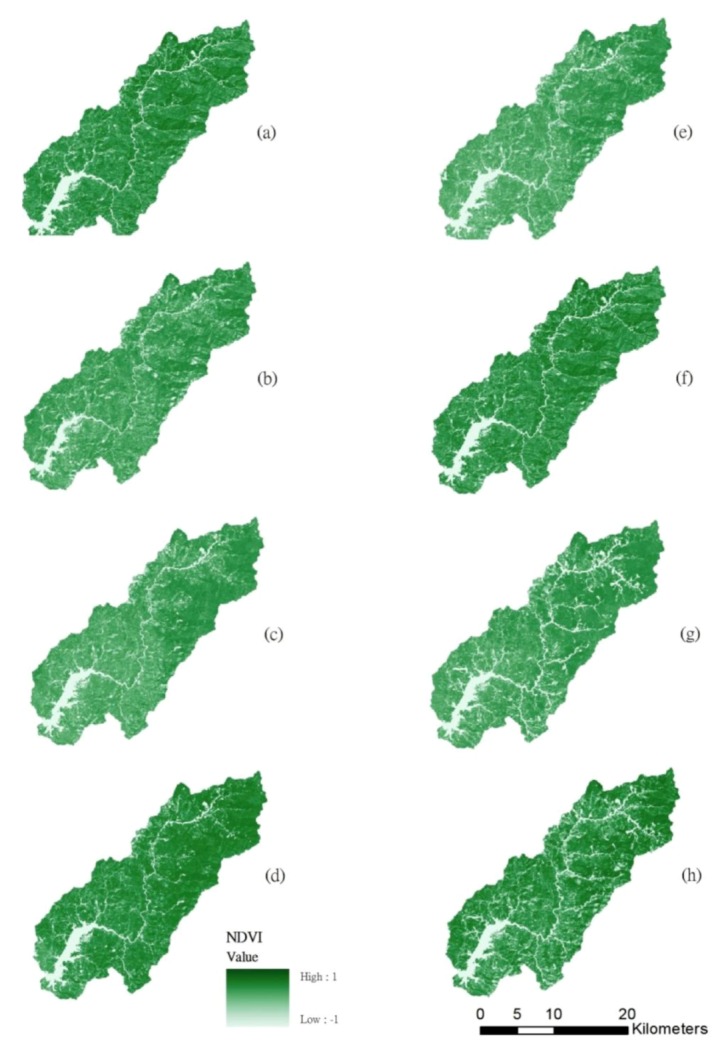
Images of NDVI patterns in the study area during (**a**) 2001, (**b**) 2003, (**c**) 2004,(**d**) 2005, (**e**) 2007, (**f**) 2008, (**g**) 2009 and (**h**) 2010.

**Figure 5:**
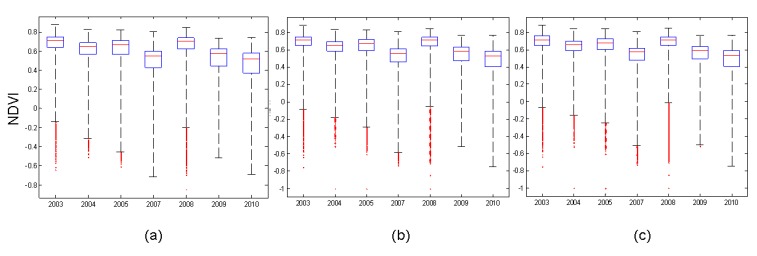
Boxplot of NDVI values for (**a**) 1, (**b**) 2, and (**c**) 3 km buffer zones.

Figure 2 shows that land use classification in 2001, 2004, 2007 and 2010. The forested land, grassland, bare land, build-up, and landslide accounted for 77.61%, 11.72%, 7.72%, 2.55% and 0.42% (excluding water) of the total watershed area in 2001, respectively. During 2001–2010, forest has decreased 4.81%, grassland has increased 2.35%, landslide has increased 2.12%, bare land has increased 0.55%, and built-up land has decreased 0.21% (Figure 3). The results matched previous studies [21,22] that many landslides in the Tseng-Wen reservoir watershed were caused by typhoons. Table 1 shows the statistics of NDVI images after typhoon events from 2001 to 2010. Results show that the lowest mean NDVI values (0.437) occurred on November 1, 2009, after Typhoon Morakot, and the second lowest NDVI values occurred on December 27, 2010 (0.494). The greatest impact on the landscape is from Typhoons Morakot and Fanapi. During the event (*i.e*., Typhoon Fanapi), the standard deviation of NDVI values was the largest. The analysis results of NDVI images (Figure 4) are sufficient to present land cover changes induced by disturbances, particularly by spatial structure, variability, and spatial correlation. The disturbances impacted the fragmentation and interspersion of the low NDVI patches and created heterogeneous patterns across the landscape within the affected area [16]. However, land cover change may be different in the spatial scales. The box plot shows that the range between the lower and upper quartiles in the NDVI decreases when the buffer zone increases (Figure 5).

### 3.2. The Change of Water Quality

Table 2 shows the correlation coefficients between the average NDVI in the watershed and average water quality factors during the whole year and after typhoon. The average value of the NDVI in the watershed is strongly correlated with NO_3_-N during the entire year and is strongly correlated with SS after typhoon from 2001 to 2010. These water quality factors such as SS and NO_3_-N concentrations are the indices for water quality assessment when considering land cover changes. The average SS concentrations after typhoons and the average annual NO_3_-N in the sites are used by later descriptive statistics and regression analysis.

**Table 2:** Correlation coefficients of average NDVI and water quality in whole year and after typhoon.

	NO_3_-N	SS	COD	DO	TP	Turbidity
**Whole year **	0.687	−0.577	0.277	−0.498	0.313	0.086
**After typhoon**	0.529	−0.621	0.604	−0.060	0.364	−0.384

NO3-N: nitrate-nitrogen; SS: suspended sediments; COD: chemical oxygen demand; DO: dissolved oxygen; TP: total phosphorus.

**Table 3:** Descriptive statistics of SS, and NO_3_-N data in three water quality-monitoring stations during 2001–2010.

		Mean	SD	Q25	Q75	Min	Max
SS (ppm)	Site 1	4.51	2.47	2.90	5.70	0.80	13.50
	Site 2	4.85	2.61	3.10	6.05	1.10	12.60
	Site 3	4.52	2.14	2.90	5.78	1.00	9.80
NO_3_-N (ppm)	Site 1	0.44	0.31	0.25	0.58	0.01	1.62
	Site 2	0.47	0.30	0.26	0.60	0.01	1.45
	Site 3	0.45	0.28	0.28	0.58	0.01	1.20

SD: standard deviation; Q25: the first quartile; Q75: the third quartile; Min: minimum; Max: maximum.

**Figure 6:**
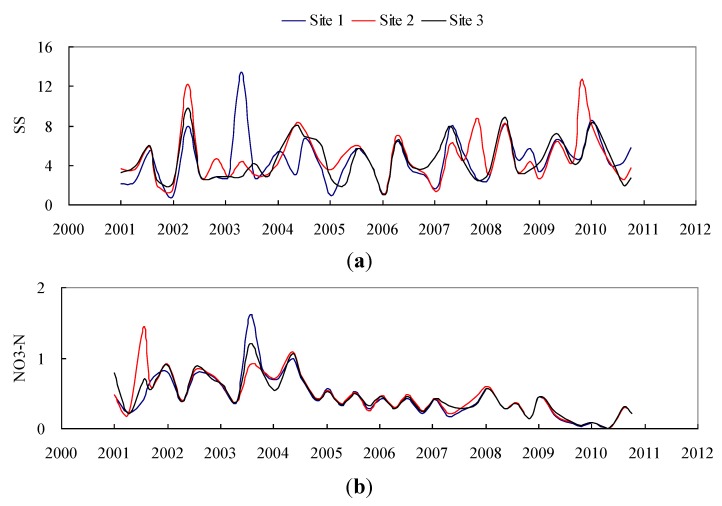
Temporal variation of (**a**) SS and (**b**) NO_3_-N during 2001 and 2010 (unit: ppm).

Table 3 lists quarterly measurements of SS, and NO_3_-N data at three water quality-monitoring stations from 2001 to 2010 (Figure 6). The average concentrations for SS for observation Sites 1, 2, and 3 are in the ranges of 0.8–13.5 (ppm), 1.1–12.6 (ppm), and 1.0–9.8 (ppm), respectively. Moreover, the average concentration of NO_3_-N for Sites 1, 2, and 3 are in the ranges of 0.01–1.62 (ppm), 0.01–1.45 (ppm), and 0.01–1.20 (ppm), respectively. The average SS values are 4.51, 4.85, and 4.52 (ppm), and the average NO_3_-N values are 0.44, 0.47, and 0.45 (ppm) in these 3 sites. Figure 6(a) shows that SS concentrations are cyclical. Typhoons and heavy rainfalls trigger large sediment discharge into the rivers of Taiwan and cause high-suspended sediment concentrations during these events*.* Most nitrate concentrations in the water drains from agricultural land. However*,* Figure 6(b) shows NO_3_-N concentrations vary with a decreasing trend.

### 3.3. Relationship between SS Concentration and Land Cover Change

Table 4 shows the regression models for water quality and land cover changes at various spatial scales. The factors comprise the average NDVI in the watershed, and the average NDVI in 1 km, 2 km, and 3 km buffer zones. Results show that annual nitrate-nitrogen concentrations are positively correlated with the NDVI with a 1 km buffer area. However, SS is negatively impacted by the average NDVI in the watershed, suggesting that typhoons impact land cover change in the watershed. For example, typhoons cause landslides, and these are a major source of soil erosion and sediment yield in the watershed*.* The average NDVI in the watershed adversely impacts the water quality, and therefore, increases sediments associated with water quality. The average NDVI in the watershed becomes a key factor influencing the SS concentration. Typhoon events are major natural disturbances causing NDVI changes and also cause serious landslides [16,23,24]. Both important factors affecting soil erosion and sediment delivery to river channels are changes in land use and climate. Due to destruction of vegetation and increased soil exposure in the watershed after rainstorms and typhoons, the NDVI values decreased. The rainstorms and typhoons cause divergent destruction of vegetation, and led to an increase in the potential for soil erosion.

**Table 4:** Regression model for the function of water quality and average NDVI in various scales.

	SS	NO_3_-N
Const.	6.86	−0.73
NDVI_Watershed	−23.74 *	-
NDVI_Buffer1	-	2.10*
NDVI_Buffer2	-	-
NDVI_Buffer3	20.23	-
*R^2^*	0.65	0.75

* represents *p* < 0.05; NDVI_Watershed: average NDVI in whole watershed; NDVI_Buffer1: average value of NDVI in 1 km buffer zone; NDVI_Buffer2: average value of NDVI in 2 km buffer zone; NDVI_Buffer3: average value of NDVI in 3 km buffer zone.

### 3.4. Relationship Between NO_3_-N Concentration and Land Cover Change

Results show that the dominant explanatory variables in NO_3_-N cases have an average NDVI with a 1 km buffer zone. In some reservoirs and lakes, the primary indicator of agriculture is dependent on NO_3_-N concentration. The nitrate concentration is correlated with agricultural practices during the high-flow period. Human activity alters the patterns of nitrate concentrations during storm events in the agricultural catchment [25]. Since Taiwan joined the World Trade Organization (WTO) in 2002, imported agricultural products are cheaper than domestic ones, thus negating the need for extensive agriculture areas. This corresponds to the data that tea farms in Chiayi County decreased from 2,292 to 2,189 ha from 2005 to 2011 [26]. Forestry, agriculture, and anthropogenic activities impact the quality of water over short and long periods [27]. The SS and NO_3_-N are typically sensitive in landslide and agriculture land areas. The previous results match that percent agricultural coverage had a significant influence on both SS and nitrate-N loading [7]. NDVI variation results imply that as land cover changes; hence, the multi-scale NDVI, which is one of the indices in the watershed of land cover changes, is associated with water quality and is hard to directly link with agriculture. However, the land use classification of SPOT images is also hard to identify the agriculture land. Further study could consider the high-resolution satellite images in the land use classification.

## 4. Conclusions

This study examined the NDVI images from 2001 to 2010 based on SPOT imagery data. The imagery shows that the land cover changes in the study vary with the influences of typhoons and human activities. Satellite image data showed a general decline in the acreage of vegetation cover implying increased landslide and decreased forest pressure on the vegetation resources.

Land cover change had a significant influence on both suspended solid and nitrate-nitrogen loadings. Simple regressions were performed that showed water quality is related to land cover in various spatial scales. Annual NO_3_-N concentration is positively correlated with an average NDVI with a reservoir 1 km buffer area, but SS are negatively correlated with an average NDVI in the watershed after typhoon events. Understanding the relationship between land cover change and water quality is useful for watershed management and pollution prevention plans. Further study should add additional spatial and independent variables to the models, such as percentage of land use type, anthropogenic activities and typhoon precipitation. 
